# In Response to: ‘Impact of Glycosylation on Effector Functions of Therapeutic IgG’ (*Pharmaceuticals* 2010*, 3,* 146–157)

**DOI:** 10.3390/ph3061887

**Published:** 2010-06-10

**Authors:** Andreas Nechansky, Iris Koller, Ralf Kircheis

**Affiliations:** 1 Meridian Biopharmaceuticals GmbH, Brunnerstrasse 59, 1230 Wien, Austria; 2 Vela Pharmazeutische Entwicklung und Laboranalytik GmbH, Brunnerstrasse 59, 1230 Wien, Austria; E-Mail: i.koller@vela-labs.at (I.K.); 3 Virologik GmbH, Henkestrasse 91, 91052 Erlangen, Germany; E-Mail: r.kircheis@virologik.com (R.K.)

**Keywords:** therapeutic antibodies, glyco-modified IgG, ADCC, CDC, FcγRIII

## Abstract

To complete the review article by Abes and colleagues (*Pharmaceuticals* **2010***, 3,* 146–157) which provides a good overview on recently developed approaches for generation of glyco-modified antibodies and the impact of glyco-modification of antibodies on their effector functions, important information should be added, namely that — besides the Glycart and the Biowa approach to generate de-fucosylated antibodies — innovative, moss derived methods have been shown to generate glyco-modified antibodies with improved effector function profile.

## 1. Introduction

The review article written by Abes *et al*. entitled ‘Impact of Glycosylation on Effector Functions of Therapeutic IgG’ [1] provides a good overview on the approaches used for generating glyco-modified antibodies, with a focus on de-fucosylated antibodies. In order to extend the information provided in this review article, important recent data should be added, namely that — besides the approaches developed by Glycart and Biowa, respectively, to generate de-fucosylated antibodies — innovative alternative methods, in particular moss derived methods, have been used to generate glyco-modified antibodies with improved effector function profile. In an approach recently developed by Greenovation, a plant derived expression system based on a gene-engineered fucosyl-transferase and xylosyl-transferase deficient moss line allows for a completely animal component-free recombinant expression of therapeutic mAbs with tailor made *N*-linked glycosylation devoid of core fucose. Glyco-modified mAbs stably expressed in this unique glyco-engineered expression system show a highly homogeneous N-glycosylation pattern lacking core-fucose. While glyco-modification of mAb does not affect the binding specificity to the target structure, effector functions such as antibody-dependent cellular cytotoxicity (ADCC) and complement-dependent cytotoxicity (CDC) can be modulated using this plant-based expression system. Another plant based expression system developed by Biolex uses the small aquatic plant Lemna as expression host. Optimized glycosylation is accomplished by co-expressing an interfering RNA (RNAi) construct targeting the endogenous α-1,3-fucosyl-transferase (FucT) and β-1,2-xylosyl-transferase (XylT) genes.

## 2. Discussion

A major limitation to the application of therapeutic monoclonal antibodies (mAbs) is their reduced *in vivo* efficacy compared to the high efficacy measured *in vitro*. Effector functions such as ADCC are dramatically reduced *in vivo* by the presence of high amounts of endogenous IgG in the serum [2]. It has been shown that the modification of the glycosylation moieties attached to the Fc part of the mAb, *i.e.* the removal of the core fucose residue, strongly enhances binding affinity to FcγRIIIα and thus might antagonize with the reduced *in vivo* efficacy [3,4]. Plant derived glyco-modified techniques such as Lemna employed by Biolex (USA) [5] or fucosyl- and xylosyl-transferase deficient, (Δxyl-t/Δfuc-t) double knock-out moss cell lines developed by Greenovation (Germany) [6] offer advantages compared to the non-plant methods like safety (no human pathogenic viruses), reduced costs (salts and light are the main sources for growth) and purification. 

**Figure 1:**
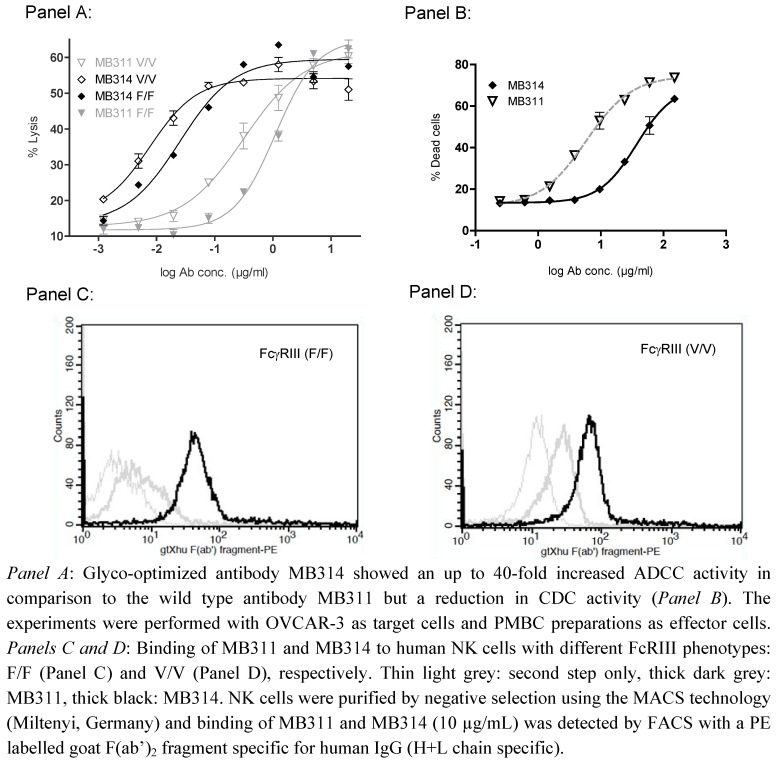
ADCC, CDC and NK cell binding by MB311 compared to its glyco-engineered variant MB314.

Regarding clinical data obtained with plant produced proteins, Biolex is currently testing Locteron for the treatment of chronic hepatitis C in a clinical Phase IIb study [7]. On the antibody field both companies, Biolex and Greenovation have published pre-clinical results that demonstrate the feasibility of two different plant-based approaches: the Biolex data highlight the ability of their LEX System to produce an anti-CD20 antibody with an optimized glycosylation structure with enhanced ADCC, more potent B-cell depletion, and potentially lower side effects compared to Rituximab, the current standard of care for the treatment of non-Hodgkin’s B-cell lymphoma [5]. The Greenovation technology was applied to engineer the humanized Lewis-Y carbohydrate recognizing monoclonal antibody MB311 which mediates tumor cell lysis via CDC and ADCC [4,6]. Beside its (low) ADCC and strong CDC effector functions, MB311 also was shown to inhibit the signal cascade of Lewis-Y glycosylated ErbB receptors [8]. MB311 has successfully completed an open-label dose escalation Phase I trial showing good safety and pharmacokinetic profile, long-lasting cytolytic activity against tumor cells in patients’ sera, and elimination of circulating tumor cells. The glyco-modified version of MB311, designated MB314, showed a highly homogeneous N-glycosylation pattern quantitatively lacking the core-fucose (and -xylose) and was compared side by side to its parental counterpart MB311 (formerly IGN311) produced in conventional mammalian SP/2 cell-culture. As confirmed by FACS and ELISA, the target specificity of MB314 was similar to that of MB311, but the ADCC effector function was increased significantly (up to 40-fold) [6]. In contrast complement-dependent cytotoxicity (CDC) activity was decreased (Figure 1). 

An increased binding affinity of the glyco-modified MB314 to the FcγRIII expressed on Natural Killer (NK) cells was found to correlate with increased ADCC activity. Interestingly, the binding to both, high affinity as well as low affinity FcγRIII was significantly enhanced for the glyco-modified antibody (Panels C and D). The data demonstrate that by different glyco-engineering approaches the effector function profile including ADCC, CDC or cytokine release can be fine-tuned according to the therapeutic needs. However, productivity is still limited in current plant expression platforms compared to the established CHO processes and also the scale up process is challenging. Such issues are typical for new technologies and will most likely be overcome within the next years. 

In summary, the potential of the de-fucosylated antibodies lies in: (i) a potentially reduced treatment dose, (ii) an increased therapeutic efficacy, and (iii) a broader therapeutic window. Consequently, these features should also allow entering into new therapeutic indications. Plant-based recombinant protein production systems offer a safe and cost-effective alternative to traditional microbial and mammalian cell culture systems.
